# Vertical GaN-on-GaN Schottky Diodes as α-Particle Radiation Sensors

**DOI:** 10.3390/mi11050519

**Published:** 2020-05-20

**Authors:** Abhinay Sandupatla, Subramaniam Arulkumaran, Ng Geok Ing, Shugo Nitta, John Kennedy, Hiroshi Amano

**Affiliations:** 1 School of Electrical and Electronics Engineering, Nanyang Technological University, Singapore 639798, Singapore; 2 Temasek Laboratories in Nanyang Technological University, Research Techno Plaza, 50 Nanyang Drive, Singapore 639798, Singapore; Subramaniam@ntu.edu.sg; 3 Center for Integrated Research of Future Electronics (CIRFE), IMaSS, Nagoya University, Nagoya 464-8603, Japan; nitta@nagoya-u.jp (S.N.); amano@nuee.nagoya-u.ac.jp (H.A.); 4 National Isotope Center, GNS Science, Lower Hutt 5010, New Zealand; J.Kennedy@gns.cri.nz

**Keywords:** GaN-on-GaN, schottky barrier diodes, high-energy α-particle detection, low voltage, thick depletion width detectors

## Abstract

Among the different semiconductors, GaN provides advantages over Si, SiC and GaAs in radiation hardness, resulting in researchers exploring the development of GaN-based radiation sensors to be used in particle physics, astronomic and nuclear science applications. Several reports have demonstrated the usefulness of GaN as an α-particle detector. Work in developing GaN-based radiation sensors are still evolving and GaN sensors have successfully detected α-particles, neutrons, ultraviolet rays, x-rays, electrons and γ-rays. This review elaborates on the design of a good radiation detector along with the state-of-the-art α-particle detectors using GaN. Successful improvement in the growth of GaN drift layers (DL) with 2 order of magnitude lower in charge carrier density (CCD) (7.6 × 10^14^/cm^3^) on low threading dislocation density (3.1 × 10^6^/cm^2^) hydride vapor phase epitaxy (HVPE) grown free-standing GaN substrate, which helped ~3 orders of magnitude lower reverse leakage current (*I_R_*) with 3-times increase of reverse breakdown voltages. The highest reverse breakdown voltage of −2400 V was also realized from Schottky barrier diodes (SBDs) on a free-standing GaN substrate with 30 μm DL. The formation of thick depletion width (DW) with low CCD resulted in improving high-energy (5.48 MeV) α-particle detection with the charge collection efficiency (CCE) of 62% even at lower bias voltages (−20 V). The detectors also detected 5.48 MeV α-particle with CCE of 100% from SBDs with 30-μm DL at −750 V.

## 1. Introduction

Nuclear reactions normally emit different high-energy particles such as α-particle, β-particles, neutrons, γ-radiation and x-rays. As each of these emitted particles interacts with matter differently, a study into α-particle detection is highly important [1]. Due to the high mass and density of an α-particle, the distance traveled by α-particle is limited to only a few centimeters during which it loses all its energy along the path. In general, α-particles traverse only a few microns in any solid before losing all its energy. The energy transferred from the α-particle gets converted into heat. This distance traversed by α-particle plays an important role as only the atoms in this area can interact with the α-particle. 

### 1.1. Gaseous Ionization Detectors 

**Figure 1:**
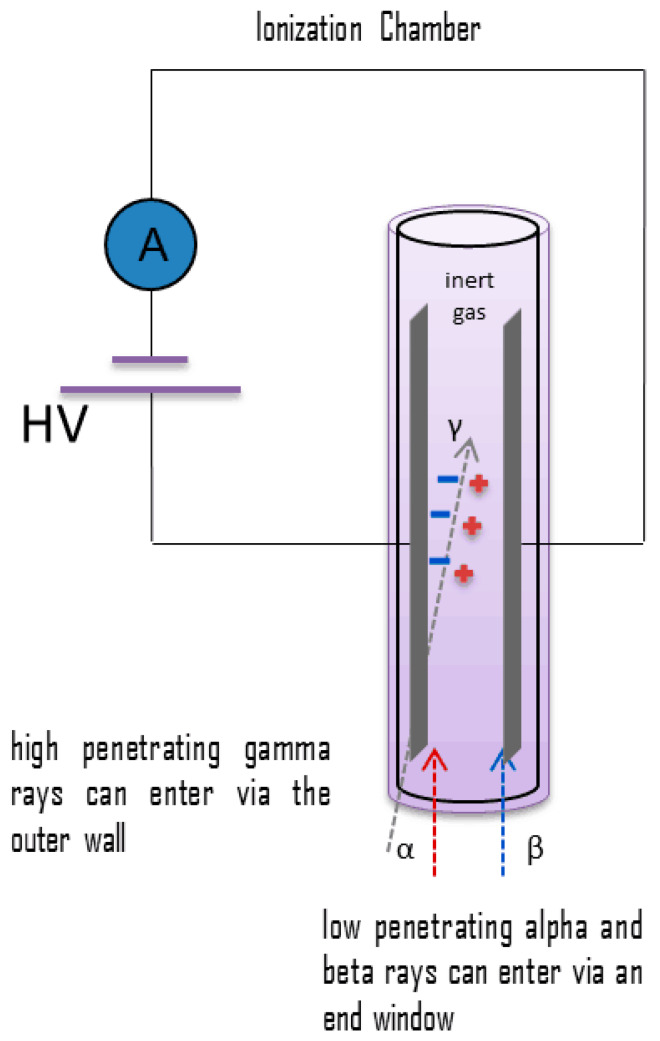
Schematic set-up of an Ionization detector [2,3].

Ionization detectors are used to detect ionizing radiation like α-particle and β-particle. Figure 1 shows a typical schematic of an ionization chamber in which an external voltage is applied to keep the conditions in ionization region. A basic ionization detector consists of a chamber filled with a suitable gaseous medium (see Figure 1). Ionization detector is dependent on the effect of a charged particle passing through the gaseous medium. The gaseous medium should have the following qualities:

- chemically stable and inert
- low ionization energy realizes maximum ionization of the medium
- low sensitivity to radiation damage to realize a longer lifetime of the detector.

Typically noble gases like helium (He) and argon (Ar) are used in nuclear power plants to measure α-particles, β-particles and γ-rays.

The ionization chamber has two electrodes across which a very large voltage (>1 kV) is held. When ionizing radiation enters the ionization chamber it generates electron-ion pairs, whose behavior is dependent on the external electric field. Under the high electric field, the generated electron-ion pairs move towards opposite electrodes as the extremely high electric field prevents their recombination. 

Ionization chambers are preferred for high radiation dose rates as they do not have any dead time as these detectors have no inherent amplification of the signal. The absence of an amplification component enables the use of ionization chamber immediately after large current detection. In addition, the absence of amplification also helps to provide excellent energy resolution as amplification increases electronic noise. 

Although Ionization chambers have many advantages discussed above, the high voltage requirement and the required directionality of incident α-particles restricts its use. The use of a gas chamber increases the fragility of the equipment and reduces portability of the detector. Further details on the principle and method of operation of ionization detectors can be found in Lamarsh, J.R. et al. [1], Burn, R.R. et al. [4] and Rossi, B. et al. [5].

### 1.2. Scintillation Detector

**Figure 2:**
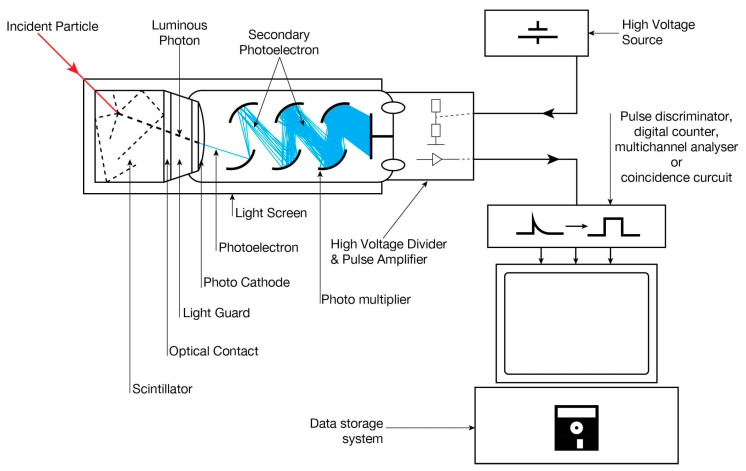
Schematic set-up of a Scintillation detector [3].

Scintillation is a flash of light observed when a transparent material interacts with a charged particle. By detecting the flashes of light produced by a scintillator using a photodetector detection of radiation is possible (see Figure 2). A scintillation detector mainly consists of two key elements.

- Scintillator—it generates photons in response to incident radiation.
- Photodetector—a sensitive photodetector converts the incident light into an electrical signal.

The basic operating principle of the scintillation detector involves the conversion of incident radiation energy to optical energy by a scintillator, which produces flashes of varying intensity. The intensity of the optical energy generated is dependent on the energy of the incident radiation (see Figure 2). 

Scintillation detectors are highly beneficial for its high efficiency, precision and counting rates. 

The intensity of flashes generated by the scintillator and the output voltage is directly proportional to the energy of the incident particle. Therefore scintillators can be used for the determination of the number of incident α-particles and their energy. 

The use of Scintillation based detector involves multiple energy conversions resulting in conversion losses and introduction of noise. Along with multiple energy conversions presence of amplifiers in the circuit results in a significant dead time which reduces the maximum dosage of α-particles which can be detected. The amplifier also introduces electronic noises resulting in poorer energy resolution. Apart from the disadvantages due to the setup, scintillation based detectors generate 1/10 the intensity of light due to the incidence of α-particles when compared electrons of the same energy. This reduced sensitivity is due to α-particles being heavier in comparison to electrons. Inorganic crystals like ZnS are generally used in the fabrication of scintillators [1,2]. Further details on the principle and method of operation of scintillation detectors can be found in Teo, W.R. et al. [6] and Knoll, G. et al. [7].

### 1.3. Solid-State Semiconductor Detectors

**Figure 3:**
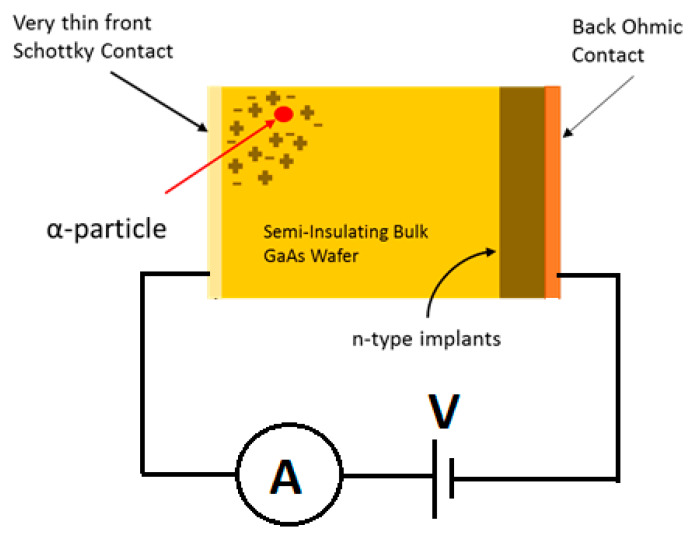
Schematic diagram of GaAs Schottky Barrier Diodes for α-particle detection.

Solid-state detectors are made of semiconductors and operate by generating current on interaction with ionizing radiation. The interaction of semiconductor material with ionizing radiation like α-particle results in the excitation of an electron. This excited electron moves out of its energy level creating electron-hole (e-h) pairs. The energy of the incident radiation particle is utilized to generate multiple e–h pairs, hence higher the incident energy higher the e-h pair generation. Figure 3 shows a schematic of a semi-insulating GaAs α-particle detector.

Operating principles of Semiconductor radiation detectors:

- Ionizing radiation enters the depletion width (DW) of Schottky Barrier Didoes.
- Radiation passing through the DW generates e-h pairs. The number of e-h pairs generated depends on the incident energy of the particle.
- The external electric field forces e-h pairs to traverse to the electrodes and result in a pulse which can be recorded by an external circuit. The ratio of detected energy to incident energy has been termed as charge collection efficiency (CCE).
- Generated pulse carries information about the energy of the incident radiation and the number of such pulses gives the intensity of the radiation.

Based on the principles of operation of semiconductor detectors, for their successful operation, the following considerations are important. These considerations can be classified into material characteristics and device characteristics.

- Material Characteristics—Material characteristics like the displacement energy (*E_d_*) and e-h pair creation energy of the semiconductor play a vital role. While *E_d_* determines the lifetime of the detector, e-h pair creation energy determines the sensitivity of the detector.
- Device characteristics—Electrical characteristics of the fabricated device like the generated DW and leakage current determine the maximum detectable energy incident on the detector and the sensitivity of the detector.

#### Properties of Selected Semiconductors 

While electrical properties like *E_d_* and e-h pair creation energy of the semiconductors play a vital role in determining the detector performance, other properties like thermal conductivity, bandgap, breakdown strength and so forth, also play a role. Bandgap defines the lowest detectable energy, thermal conductivity regulates the maximum operable temperature range and breakdown strength determines the maximum DW, which can be generated for any material. A summary of important material characteristics has been listed in Table 1 below.

Si-based radiation detectors—Si has a decently high *E_d_* of 13 eV accompanied by a well-developed device fabrication technology [8]. While high *E_d_* results in a good life-time of the detector, the established fabrication technology is important to fabricate detectors with wide DW and low leakage currents. Diodes are made from narrow strips of Si (~100μm), which are then reverse biased to generate a thick DW. As α-particles pass through this DW, they cause small ionization currents that are detected and measured. Arranging multiple such thin detectors in an array can provide an accurate picture of the α-particle distribution in a measurement setup. Such strip detectors are widely used in the Inner Tracking System (ITS) of A Large Ion Collider Experiment (ALICE) [3]. The matured Si technologies have resulted in the development of Si detectors with the lowest energy resolution (0.23%) [12] which is very advantageous in differentiating various spectra of α-particles based on their energies.

Similarly, other semiconductors like GaAs were also explored as an alternative to Si-based detectors. Although GaAs has lower *E_d_* at 10 eV [8], GaAs is a direct energy bandgap semiconductor that allows direct transition of electrons from the valence band to conduction band without any change in their momentum. Electrons in the direct conduction band valley experience very high mobility (~7000 cm^2^/V·s) which helps the device function at lower voltages. The high electron mobility in GaAs accompanied with developed growth process development of GaAs have led to the development of Semi-insulating GaAs based α-particle detectors. The low doping density helps generate a thick DW at even at low voltages resulting in decently good energy resolution (0.89%) [17]. The primary drawback of both GaAs and Si-based detectors is that they have low *E_d_* resulting in a lower lifetime of the detector.

Although SiC, GaN and Diamond are not as developed in terms of growth and fabrication, their material characteristics exhibit their immense potential as radiation detectors due to their higher *E_d_*. Among the semiconductors listed in the Table 1, diamond shows the best material characteristics for radiation detection [16], which has resulted in the fabrication of α-particle detectors with a high energy resolution of 0.35% while detecting 5.48 MeV α-particles with 100% CCE at 15 V. Despite the superior material characteristics, the difficulties involved with the growth of a single crystalline diamond accompanied by the cost involved restricts the usage of single crystalline diamond for radiation detection. While SiC based α-particle detector have performed exceedingly well in low voltages with good energy resolution (0.25%), the *E_d_* of SiC is still lower than GaN making GaN a better choice for fabricating α-particle detectors.

## 2. GaN α-Particle Detector 

Among the III-V semiconductors, Gallium Nitride (GaN) emerged as the best semiconductor materials for lighting [18,19,20], electronic [21,22] and sensing applications [23,24] due to their superior inherent material properties such as a high direct bandgap, critical electric field, electron and saturation velocity in comparison with other popular semiconductors. High energy bandgap accompanied with large theoretical *E_d_* (109 eV for N and 45 eV for Ga) [25] and high thermal stability (melting point 3500 K at a 9 GPa pressure [26]) has also resulted in GaN being used for radiation detection applications [27]. Compared with semiconductor materials like Si and GaAs, GaN can operate at higher temperatures for a longer time. A review article by Sellin, P.J. in 2006 compared different wide bandgap semiconductors in high radiation environments and concluded that GaN was a promising candidate for α-particle detection despite GaN being relatively immature as a semiconductor [28]. 

The first group to report an α-particle detector fabricated on GaN employed a 2–2.5 μm thick GaN layer grown by metalorganic chemical vapor deposition (MOCVD) on a sapphire substrate. While these detectors performed reasonably well, their performance was highly limited due to the thin DW and high leakage currents in the devices. The absence of free-standing GaN substrate resulted in hetero-epitaxial GaN which used sapphire, Si or SiC as its substrates. The high lattice mismatch between epitaxial GaN with its substrate has resulted in high threading dislocation density (TDD). High TDD increases the reverse leakage current (*J_R_*), which is detrimental to the α-particle detector performance.

With the improvement in GaN growth technology researchers developed free-standing GaN with low TDD and thereby GaN-on-GaN wafers. This led to the development of Schottky barrier diodes (SBD) which have thick epitaxial layer and low TDD. Zhao et al. has previously reported the effects of reduced TDD by comparing SBD characteristics of GaN on sapphire and GaN-on-GaN SBDs. Use of GaN substrate has helped reduce TDD by ~3 orders of magnitude (see Table 2) thereby reduce leakage current by more than ~6 orders of magnitude [29].

In addition to the semiconductor material properties, the electrical properties of DW also affect the performance of the detector. Devices like p-n diode, pin diode and Schottky diode structures can be used to generate a DW. Detector performance widely depends on the device fabricated. 

### 2.1. p-n Diodes

**Figure 4:**
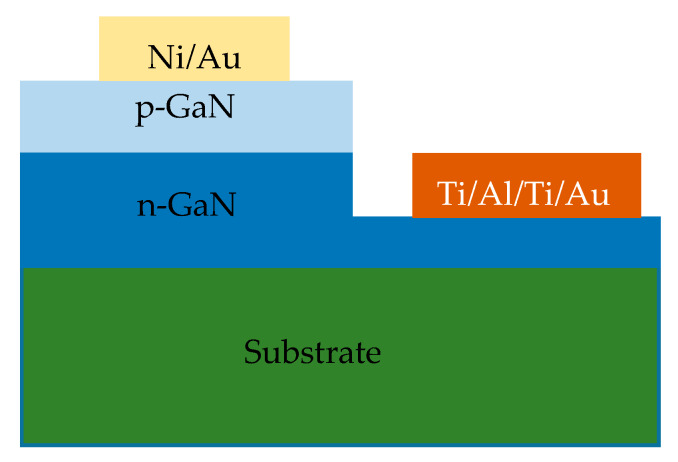
Cross-sectional schematic of GaN p-n diodes for α-particle detectors.

Sugiura, M. et al. has recently reported for the first time a p-n diode-based α-particle detector. Figure 4 shows the schematic of GaN p-n diode used for α-particle detection [30]. These p-n diodes exhibited a mobility/life-time product of 4.6 × 10^−5^ cm^2^/V which is lower than the values reported for CdTe (~10^−3^ cm^2^/V) and TlBr (~10^−3^ cm^2^/V). From the values of mobility/life-time product M. Sugiura et al. also concluded that GaN is a suitable material for radiation sensing applications.

### 2.2. PIN Diodes

Wang, G. et al. has reported a PIN diode based α-particle detector. The use of an 8 μm intrinsic GaN layer between p-GaN and n-GaN increases the thickness of formed DW, which in turn helps to detect higher energy particles with improved sensitivities [31]. 

**Figure 5:**
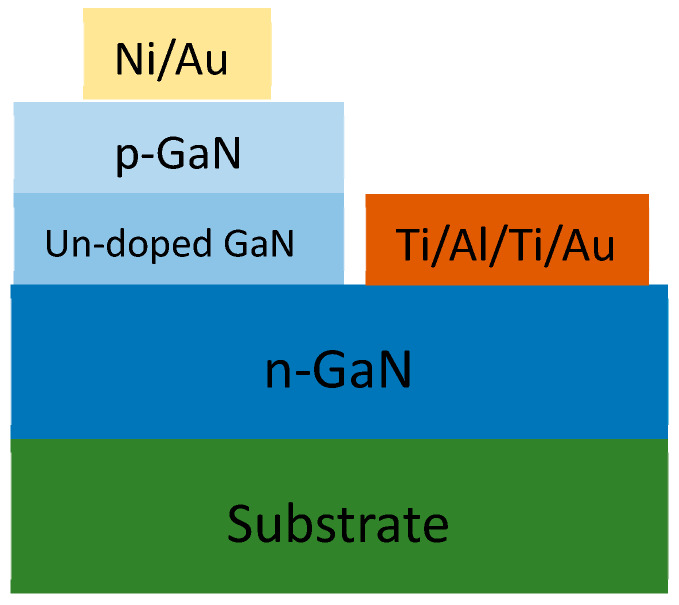
Cross-sectional schematic of GaN PIN diode structure for α-particle detector.

Figure 5 shows the diode schematic of the PIN α-particle detector used to detect 700 keV α-particles with a CCE of 80% and an energy resolution of 50%.

Authors also predict the use of a thicker intrinsic layer may increase detectable energy and reduce energy resolution.

### 2.3. Schottky Barrier Diodes

Unlike the p-n diodes and PIN diodes, SBDs have been the most popular GaN device for radiation detection. Multiple research groups have fabricated different structures at different stages of the development of GaN growth technologies. The various schematics of different structures of GaN SBD based α-particle detectors have been reviewed by Wang, J. et al. [32].

**Figure 6:**
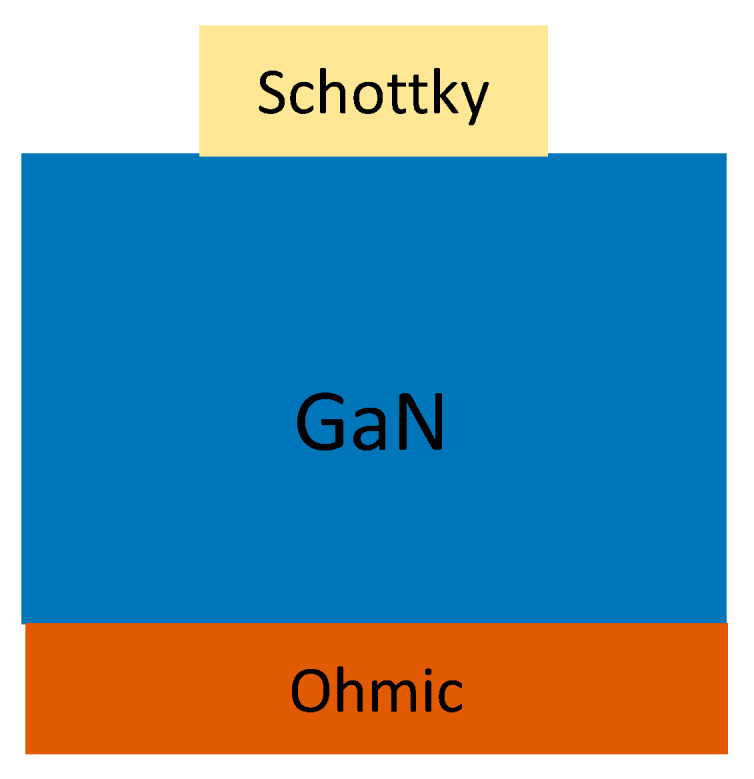
Sandwich structure of alpha particle detectors.

The best performing α-particle detector structure is shown in Figure 6. This kind of structure is called a sandwich structure. The use of a thick GaN layer sandwiched between both electrodes helps generate a thick DW hence detect higher energies of α-particles. This structure was first employed by Lee et al. [33] reported the first implementation of a sandwich structure, in which the GaN layer had unintentional H5 traps in the top 30 μm of the active area. These H5 traps resulted in reducing the charge carrier density (CCD) resulting in the detection of 5.1 MeV α-particle energy with 90% CCE. The sandwich structure was also fabricated by Mulligan et al. [34] but the detector had a very high CCD (10^16^/cm^3^) resulting in the formation of a thin DW and detection of only 325 keV α-particles. Most recently, Xu, Q. et al. has reported α-particle detector based on a sandwich structure that can detect 5.48 MeV α-particles with 100% CCE at −550 V [35], which is the highest detected α-particle energy. 

In comparison to the thin film structures, sandwich structures with free-standing GaN substrates have lower TDD. surface morphology Thicker DW helps to detect higher energy particles. Other than TDD, CCD also plays an important role in the generation of a thick DW. Higher CCD reduces DW, hence it is mandatory to reduce the CCD of the detector to detect higher energies. The use of a bulk GaN substrate with low CCD increases the resistance of the detector thereby increasing the voltage required to function at full potential. Table 3 lists all reported GaN α-particle detectors including their structure, detected energy and CCE.

From Table 3, it can be observed that higher energy has been detected by sandwich structures but they require high reverse voltage bias conditions for successful operation. To overcome the high-voltage requirement of a sandwich structure, a low CCD epitaxial layer on highly doped GaN substrate could be used [15]. The low CCD epitaxial layer helps to operate the detector at lower voltages however, highly doped substrate helps to form a low resistance Ohmic contacts.

## 3. Design Considerations and Material Characteristics

In order to improve the low voltage functionality of a GaN-based α-particle detector, an epitaxial layer whose thickness is corresponding to the target detectable α-particle is required. The required thickness of DW can be simulated using stopping range of ions in matter (SRIM) [39]. 

**Figure 7:**
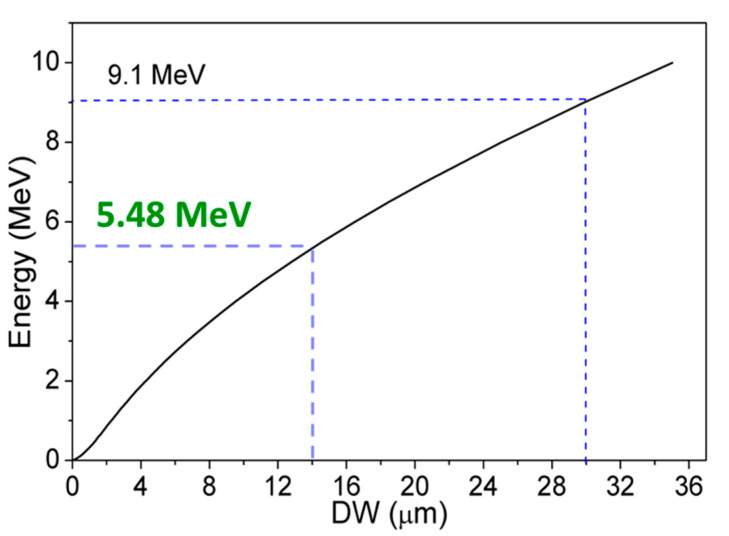
α-particle range in GaN calculated by stopping range of ions in matter (SRIM).

From the simulation results shown in Figure 7, 14.58 μm was determined to be the minimum DW required to detect 5.48 MeV α-particle energy generated from a ^241^Am source. In order to generate a 14.58 um DW SBDs with 15 μm and 30 μm drift layer thickness with very low CCD need to be fabricated.

**Figure 8:**
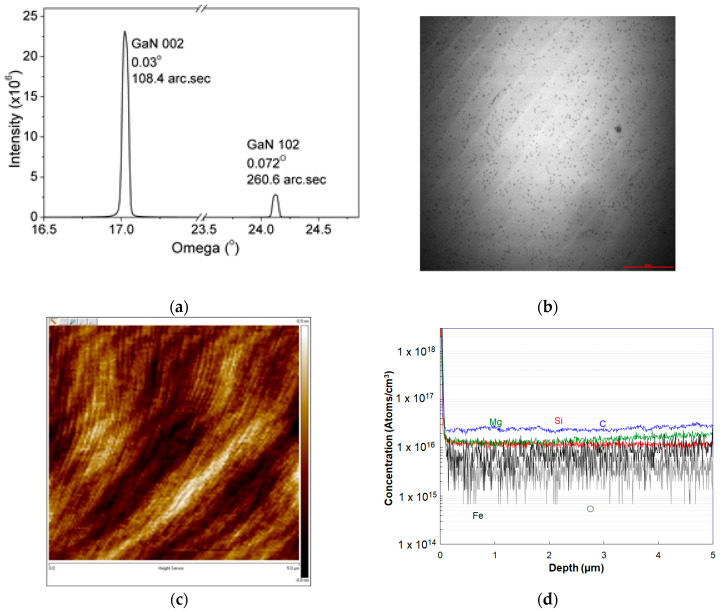
(**a**) X-ray diffraction (XRD), (**b**) TDD (MPPL), (**c**) Atomic force microscopy (AFM) and (**d**) SIMS Characteristics of the wafer.

Material characteristics of the GaN DL like crystalline quality, threading dislocation density (TDD), surface morphology and CCD of GaN DL play an important role in determining the detector performance. Use of thick GaN substrate to grow the 15 μm and 30 μm GaN DL has ensured the high crystalline quality of the DL which was measured by 2 theta-omega scan using XRD. The full-wave half maximum (FWHM) was measured at 108.4 arc.sec and 260.6 arc.sec in 002 and 102 orientations, respectively (see Figure 8a and Table 4). These values of measured FWHM are lower than the maximum reported values of 310 arc.sec (002) and 350 arc.sec (102) [40]. The use of a GaN substrate has also reduced the TDD in DL generated due to lattice mismatch between the substrate and the DL. A TDD of 3.6 × 10^6^/cm^2^ was measured using multiphoton excitation photoluminescence microscopy (MPPL) [41] which was similar to the TDD of the GaN substrate (see Figure 8b and Table 4). Polishing of the DL has reduced the rms roughness of the surface of the DL 0.206 nm (see Figure 8c and Table 4). While to reduce the unintentional n-type CCD and increase DW p-type dopant (Mg) was doped in the drift layer [42,43]. The presence of Mg and its concentration in the DL was extracted from Secondary Ions Mass Spectroscopy (SIMS) analysis. The reduced CCD of 7.6 × 10^14^/cm^3^ was measured from the elemental concentrations and verified Hall measurements (see Figure 8d and Table 4). 

## 4. Detector Fabrication

**Figure 9:**
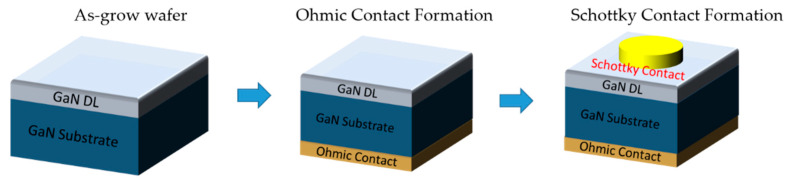
Fabrication of 1 mm GaN Schottky barrier diodes (SBD).

The SBD fabrication started with a complete cleaning of the wafer with piranha solution (H_2_SO_4_:H_2_O_2_ = 4:1) and organic cleaning (acetone and isopropanol) followed by dipping the wafer in buffered oxide etchant (BOE) for 2 min for the formation of an excellent metal-semiconductor interface [44]. After the preparation of the surface, the ohmic contact was formed by depositing Ti/Al/Ni/Au (20/120/40/50 nm) at the bottom of the wafer (N-face) of the wafer using e-beam, followed by rapid thermal annealing at 775 °C for 30 s in N_2_ ambience. Ti acts as the first layer of Ohmic stack which forms a low-resistance contact, as Ti helps in the generation of N-vacancies after annealing, which increases CCD and promotes tunneling [45]. The second layer deposited was Al which is used to absorb excessive Ti material [44], while Ni is used as a barrier metal, which confines the downward diffusion of the fourth layer (Au) [46]. The top layer of Au protects layers below from oxidization [47]. Multiple SBDs of varying sizes were then fabricated by depositing Ni/Au (50/1000 nm) on the Ga-face of the wafer. Ni was selected to be the first layer due to the difference in work functions of GaN (4.2 eV) and Ni (5.04 eV) [48], which helps to form good Schottky contact (see Figure 9). 

## 5. Electrical Characterization of SBD

To understand the effects of Mg-compensation on the performance of the SBDs, the electrical characteristics of an Mg-compensated SBDs with 2 different DL thicknesses (15 μm and 30 μm). 

### 5.1. I-V Characterization 

**Figure 10:**
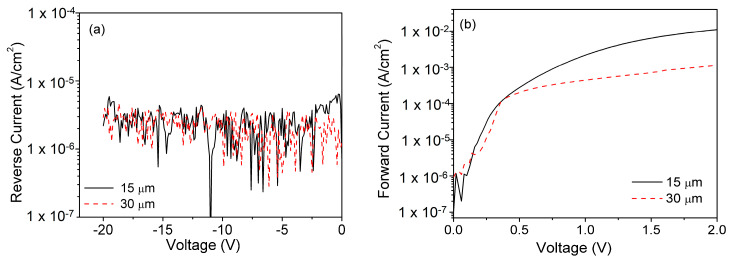
(**a**) Reverse and (**b**) Forward *I-V* characteristics of SBDs with 15 μm and 30 μm GaN DL.

Figure 10a and b show both the reverse and forward current characteristics of SBDs with 15 μm and 30 μm. It can be observed in Figure 10a that both SBDs exhibit similar *I_R_* at −20 V. Figure 10b shows the forward characteristics in which we observe a slight decrease in forward saturation current (*I_sat_*), the drop observed could be due to increase in DL thickness. Increased series resistance due to an increase in DL is the primary cause of the decrease in *I_sat_*. 

From the measured forward I-V characteristics, Ideality Factor (n) and Barrier Height (Φ_*B*_) were extracted using Equations (1) and (2) [2].

Φ_*B*_ = *KTqlnI_sat_AA∗T^2^* (1)

*I* = (*eqVnKT*/−1), (2)

where *I_sat_* is the forward saturation current, *A* is the SBD contact area, K is the Boltzmann’s constant and A* is the Richardson’s constant with a theoretical value of 26.9 A/cm^2^.K^2^. 

Average ideality factors of 1.03 and 1.05 were extracted from 10 SBDs with compensated and conventional DL, respectively. The near-unity ideality factor signifies an excellent metal-semiconductor interface at the Schottky-semiconductor contact. The use of the Piranha solution followed by organic cleaning and dipping in BOE for 2 min has resulted in the formation of an excellent metal-semiconductor interface. The similarity of values in n among the SBDs with both 15 μm and 30 μm DLs indicates the thickness of DLs does not play any role in the determination of n [49]. Similarly, the extracted Φ_*B*_ for both SBDs of 0.81 eV (15 μm) and 0.78 eV (30 μm) SBDs are close to each other and similar to other reported Φ_*B*_ for Ni-based Schottky contacts (see Table 5). From the comparison, the extracted n and Φ_*B*_ were found to be within the reported range of 1.01 to 1.4 for the ideality factor and 0.74 eV to 1.1 eV for barrier height.

### 5.2. Capacitance–Voltage (C–V) Characteristics

**Figure 11:**
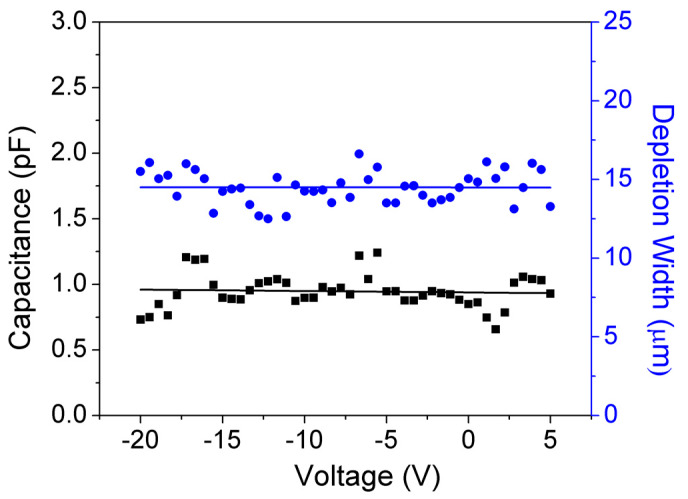
Variation of capacitance and DW with voltage of 0.5 mm diameter GaN SBDs with 15 µm.

C–V measurements were performed to extract the DW of the SBDs. No significant variation in capacitance was observed in a voltage range of −20 V to 5 V (see Figure 11), which signifies the complete depletion of the DL [55,56].

DW can be extracted from the C–V characteristics using Equation (3):

(3) $$C=\varepsilon_{0}\varepsilon_{r}\left(A/DW\right).$$

A uniform DW of ~15 μm was measured at all voltages (−20 V to 5 V), which implies the total depletion of DL even at 0 V.

### 5.3. Reverse Conduction Mechanism of SBD 

**Figure 12:**
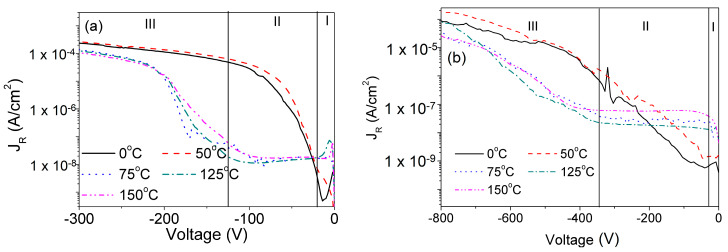
I-V-T characteristics of vertical GaN SBDs with (**a**) 15 µm DL, (**b**) 30 µm DL. Adapted from [58] A. Sandupatla et al. 2020 Appl. Phys. Express in press https://doi.org/10.35848/1882-0786/ab93a0. Copyright [2020] by Japanese Society of Applied Physics.

Reverse Conduction mechanism (CM) helps to understand the physical constituents leading to the reverse leakage current (*J_R_*) Thermionic Emission (TE) is present if all electrons traverse over the barrier and Thermionic Field Emission (TFE) is the dominant CM if electrons tunneling through the barrier. The study of CM at elevated temperatures is worthy and important to understand the overall performance of the fabricated SBDs. Moreover, high voltages are required to generate a thick DW which will help in the detection of higher energies with improved sensitivity and higher CCE [15,35]. CM of *J_R_* was extracted by comparing measured *J_R_* with theoretically calculated *J_R_* using equations for TE and TFE. Further details on the extraction of CM have been reported [43,57]. Figure 12 shows the measured *J_R_* at different temperatures over a wide voltage range for SBDs with (a) 15 μm DL and (b) 30 μm DL. I-V-T characteristics of both devices have been divided into 3 zones depending on the observed CM. 

**Figure 13:**
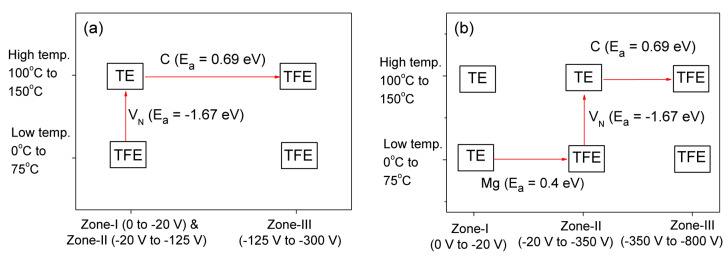
Change of CM with voltage zones and temperature ranges in SBDs with (**a**) 15 µm DL and (**b**) 30 µm DL. Adapted from [58] A. Sandupatla et al. 2020 Appl. Phys. Exp. https://doi.org/10.35848/1882-0786/ab93a0. Copyright [2020] by Japanese Society of Applied Physics.

Various changes in CM were observed with a change in both temperature and voltage, which have been shown in Figure 13. To understand the physical significance of the change in CM The activation energy was also extracted in all voltage zones [55,56]. The extracted E_a_ of 0.4 eV corresponds to the presence of Mg ions in the DL [59,60]. The activation of N-vacancies [61] (*E_a_* = −1.67 eV) with an increase in temperature resulted in the trapping of tunneling electrons and changing the CM from TFE to TE in Zone-II of both SBDs. Similarly, activation of C-traps (*E_a_* = 0.69 eV) released electrons into the depletion region when a high reverse voltage was supplied. The release of these electrons increased the probability of tunneling through the DW changing the CM to TFE. This change of CM at elevated temperatures can be used in aid of the design of high breakdown voltage SBDs for high-power switching and high-energy radiation sensing applications.

### 5.4. Breakdown Voltage of SBD

**Figure 14:**
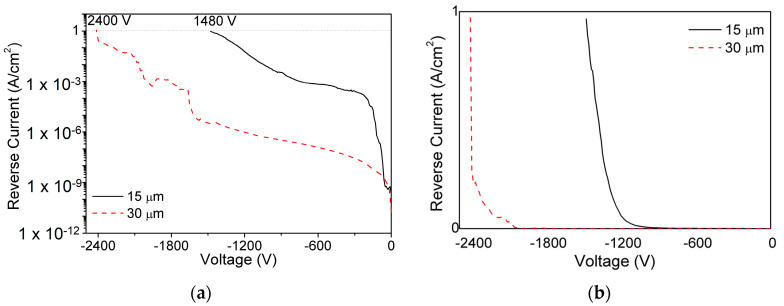
Reverse breakdown voltage characteristics of SBDs with 15 μm and 30 μm in (**a**) log and (**b**) linear scale.

The maximum voltage and power handling capability of SBDs are determined by its breakdown voltage characteristics. High voltages are essential to generate thick DW which is a primary requirement for an improved radiation detector performance. GaN has a high bandgap and high electric field strength, which makes it an optimum material to fabricate devices with high V_BD_. For breakdown characterization, SBDs were exposed to increasing voltages until it reaches the set compliance of 1 A/cm^2^ or when it reaches the catastrophic failure of the device. The SBDs were also dipped in Flourinert FC-40 prior to the measurements to insulate the SBDs from atmospheric flashover [62]. Figure 14 shows the semi-log breakdown characteristics of fabricated vertical SBDs with both 15 μm and 30 μm DLs. Thicker DW in SBDs with 30 μm DLs results in larger V_BD_. 

**Figure 15:**
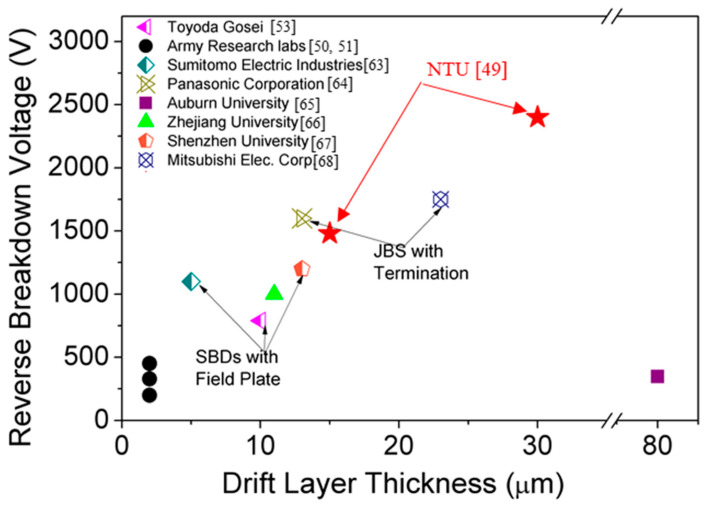
Benchmarking of measured reverse breakdown voltages of vertical GaN SBDs with state-of-the-art results.

Figure 15 shows the plot of V_BD_ vs. DLT for the state-of-the-art reported vertical SBDs [63,64,65,66,67,68]. Saitoh et al. realized a V_BD_ of 1100 V for the SBDs with DLT of 5 μm after using a field plate (FP) [63]. Shibata, D. et al. has reported the use of junction barrier Schottky (JBS) with p-type termination on SBDs with 13 μm thick DL to measure a V_BD_ value of 1600 V [64]. The improvement in V_BD_ was reported to be due to the reduction of CCD in the MOCVD grown GaN DLs [43,58].

## 6. Measurement Setup for α-Particle Detection

**Figure 16:**
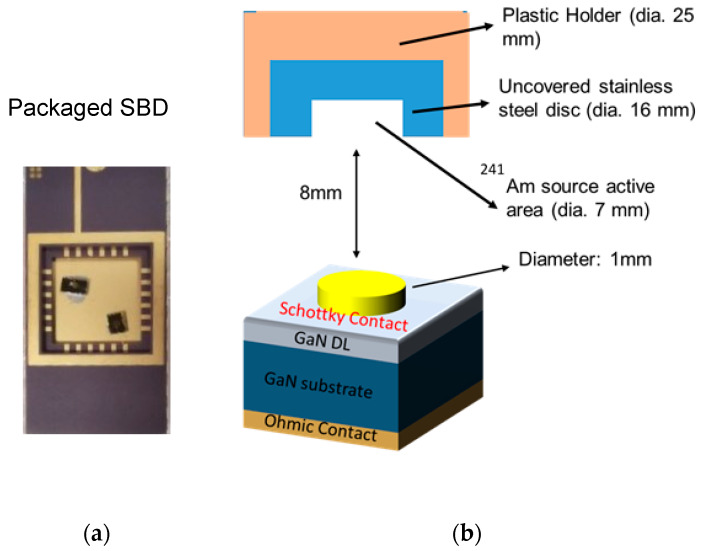
(**a**) Packaged Device and (**b**) Schematic drawing of Source-Detector measurement setup (not to scale).

After electrical characterization, both wafers were diced into individual detectors and packaged onto a dual inline package (DIP) with silver paste for the ground contact (cathode) and wire-bonding for the Schottky contact (anode) (see Figure 16a). 5.48 MeV α-particles were generated from ^241^Am source with an active area of 7 mm^2^, which was placed at 8 mm from the detector (as shown in Figure 16b). Radionuclides of ^241^Am were deposited onto a stainless-steel disc of 16 mm diameter, which was held in place by a plastic holder. The change in the current flowing through the circuit due to interaction with an α-particle was amplified by passing through pre-amplifier, amplifier and signal processing circuit. A Si surface detector from ORTEC was used as a reference, along with an ORTEC-671 amplifier for energy calibration. 

### 6.1. Detection of α-Particle Spectra 

The performance of any α-particle detectors is defined by its CCE. CCE is defined as the ratio of energy detected and the energy incident on the detector. The acquired data needs to be calibrated using a standard Si detector as a reference [69,70]. The fabricated GaN detectors require higher energy for the generation of e-h pairs in comparison with the reference Si detectors. The final detected energy is described by the following Equation (4) [34]:

*E* = *E*_0_ + *W*_GaN_/*W*_Si_ × *k* × Channel, (4)

where *E* is the energy absorbed, *E*_0_ is the loss in energy at the metal-semiconductor interface, which can be estimated from Transport of Ions in Matter (TRIM) simulations; *k* is a calibration factor of the reference detector; *W*_GaN_ is 8.9 eV and *W*_Si_ is 3.6 eV.

#### 6.1.1. Low Voltage α-Particle Detection 

For the detection of high energy α-particles, researchers have increased the DW of the detectors by fabricating them on GaN substrates. These detectors have generated 27 μm of the depleted region at very high voltages (−550 V) [34]. The requirement of high voltages in the generation of a thick DW increases the detector complexity and size, which severely affects its portability. 

**Figure 17:**
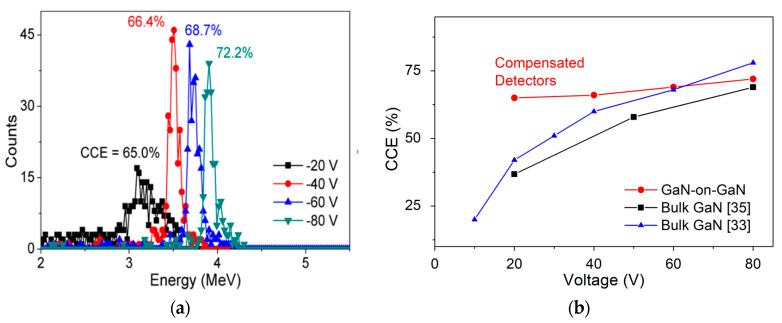
(**a**) Acquired α-particle spectra of compensated detectors for different voltages (−20 V to −80 V) and (**b**) Comparison of variation in CCE with voltages (−20 V to −80V) for state-of-the-art α-particle detectors.

The α-particle energy spectra obtained from the 15 μm detectors under low-bias conditions (−20 to −80 V) are shown in Figure 17a. Figure 17b compares the variation of CCE with the voltages of different reported detectors (sandwich structures). Detectors with compensated DL exhibited lower variation in CCE (7%) in comparison to reports using a bulk GaN-based sandwich detector. The observation of lower variation in CCE was reported to be due to formation of a thick DW.

#### 6.1.2. High Voltage α-Particle Detection 

**Figure 18:**
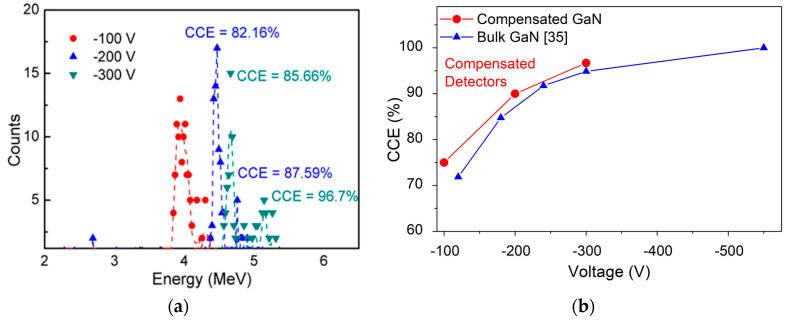
(**a**) α-particle spectra of compensated GaN detectors for different applied voltages (−100 V to −300 V) and (**b**) Comparison of variation in CCE with voltages (−100 V to −550 V) for state-of-the-art α-particle detectors.

Although researchers have increased the energy detected by using bulk GaN-based sandwich structures, the complexity of generating a thick DW only two research groups have successfully developed α-particle detectors capable of detecting 5.48 MeV energy generated from ^241^Am source. CCE of the compensated detectors improved from 72% at −80 V to 96.7% at −300 V (see Figure 18a) due to the increase in DW. The high-voltage performance of the compensated detectors requires 250 V lower bias conditions in comparison to the detector fabricated by Q. Xu et al. (see Figure 18b).

**Figure 19:**
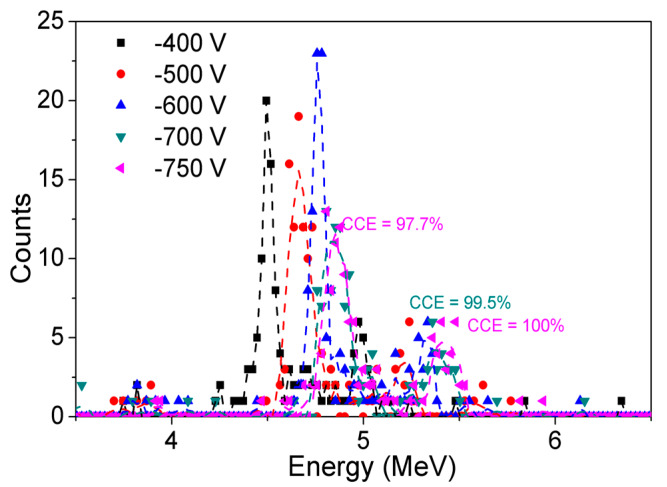
Acquired α-particle energy spectra of GaN SBDs at different voltages in vacuum.

Figure 19 shows the α-particle energy spectra obtained by SBD detectors with 30 μm DL at different voltages (−400 V to −750 V). An increase in applied bias conditions increases the detected energy increasing CCE. CCE of 100% in the detection of 5.48 MeV α-particle was obtained at −750 V. The high CCE obtained ensures complete energy detection from incident charged α-particle.

#### 6.1.3. Variation in α-Particle Spectra-Air vs. Vacuum (SBDs with 15 µm DL)

**Figure 20:**
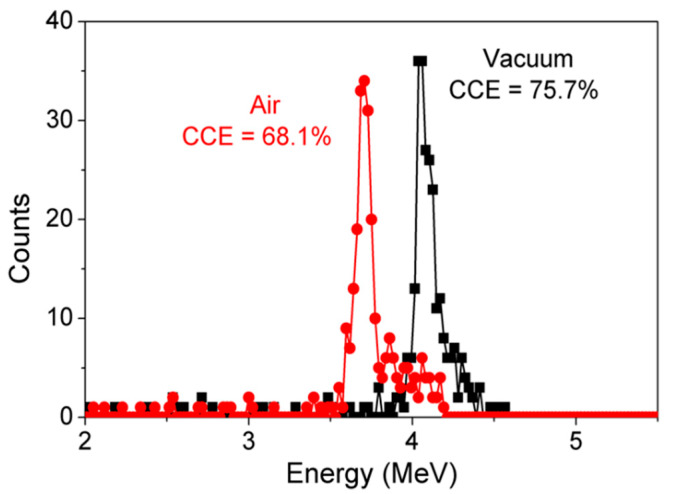
Acquired α-particle energy spectra of GaN SBDs at −100 V under air and in a vacuum.

Figure 20 shows the comparison of the energy spectrum of GaN detectors biased at −100 V measured in a vacuum and in air reported for compensated detectors. 7% reduction in CCE was reported due to the presence of air. In vacuum, the complete energy of an α-particle is transferred to the detector, resulting in the detection of higher energies. While in air energy of α-particles is lost due to scattering. This loss in α-particle energy lowers detected CCE. 

### 6.2. Benchmarking

**Figure 21:**
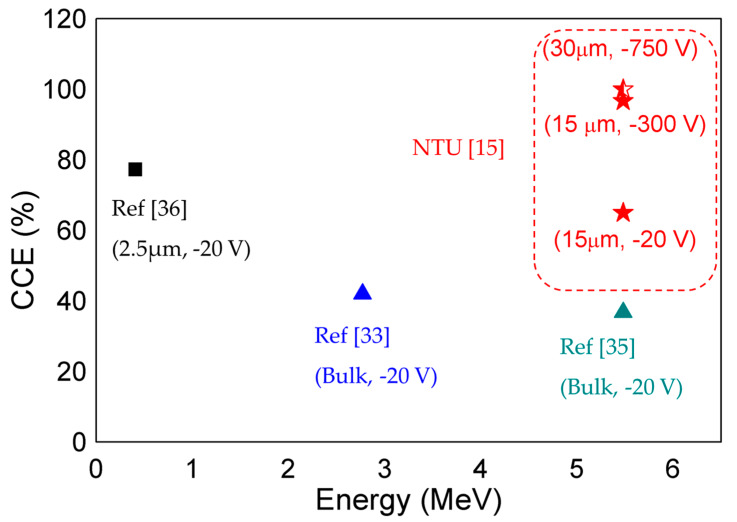
Benchmarking of extracted CCE of compensated detectors with epitaxial-grown GaN detectors (squares) and bulk GaN detectors (triangles) at low voltages.

Figure 21 compares the performance of various reported GaN-based α-particle detectors as a function of detected energies. About 30% higher CCE was reported for the compensated 15 µm α-particle detectors at −20 V. In addition, compensated detectors also exhibit 96.7% CCE at −300 V, which is 250 V in comparison to other published literature. These promising results pave the way for compensated α-particle detectors to achieve high CCE with low operating voltage.

## 7. Summary

GaN SBDs have demonstrated great potential in radiation detection on the virtue of its high displacement energy, wide bandgap and critical electric field strength. Conventionally, GaN particle detectors employ either a thin GaN epitaxial layer on the hetero-epitaxial substrate or thick free-standing GaN substrate to fabricate a radiation detector. While the thin epi-layer detector worked at low voltages (−28 V) with high CCE, they are only able to detect very low energies (< 1 MeV). The defects present in GaN such as high TDD and unintentional doping, have been the major constraints in terms of improving the detector performance. With improvement in growth technologies, free-standing GaN detectors with low TDD and doping densities have been fabricated which has increased the detected energy (5.48 MeV) but they require very high voltages (−550 V) to detect these energies with 100% CCE. 

High performing GaN α-particle detector can be achieved by designing a thin compensated GaN DL on conducting GaN substrate. Compensation of thin DL by doping p-type (Mg) ions in DL reduces the CCD generated by unintentional n-type doping. The increase in DW can reduce the J_R_ and increased the V_BD_ without affecting the ideality factor and barrier height of the SBDs. A ~3 times improvement in V_BD_ from 430 V to 1480 V due to compensation has helped realize SBDs with the highest reported V_BD_ at 2400 V (SBDs with 30 μm DL) without any additional termination or field plates.

The improvement in reverse characteristics have helped in improvement of the performance of the detector by improving the sensitivity and reducing the voltage requirement of the detector. These compensated GaN-on-GaN SBDs exhibit a 30% increase in CCE (65%) at low voltages (−20 V) in comparison to previously reported GaN α-particle detectors. High CCE of 96.7% was also measured at −300 V, which is 250 V lower than the previously reported bias requirement. The improved performance in α-particle detection is due to the formation of thicker DW at low voltages. The spectral resolution of 71 keV is also 30% better than the previous reports. Presence of air results in scattering of α-particle incident on the detector, which in turn reduced its efficiency by ~7%. While detectors with thicker DW do require a higher voltage (750 V) to detect α-particles with 100% CCE, they can also detect higher energies (9.1 MeV).

## Figures and Tables

**Table 1:** Material characteristics of different semiconductors used for the α-particle detection along with the best-reported detector performances.

Properties	Si	GaAs	SiC	GaN	Diamond
Band gap (eV)	1.12	1.4	3.3	3.4	6
Electron Mobility (cm^2^/V·s)	1450	8500	800–1000	1000	1800–2200
Sat. elec. Drift velocity (×10^7^ cm/s)	1.0	1.2	2	2	2.7
Breakdown Strength (MV/cm)	0.5	0.4	2.2	3.3	10
e-h pair creation (eV)	3.6	4.3	7.8	8.9	13
Displacement Energy, *E_d_* (eV) [8]	13	10	C-20 Si-35 [9]	Ga-45 N-109 [10]	35 [11]
Bias Voltage Required to achieve 100% CCE (V)	24 [12]	150 [13]	200 [14]	300 [15]	15 [16]
Detected Spectral Energy Resolution (%)	0.23	15	0.25	1.29	0.35

**Table 2:** Lowest reported threading dislocation density (TDD) in GaN drift layers on different substrates.

Parameter	Si	Sapphire	SiC	GaN
Lattice mismatch	−17%	−33%	3.5%	0
Thermal Mismatch	116%	−23%	24%	0
TDD (/cm^2^)	~10^9^	~10^9^	~10^7^	~10^4^

**Table 3:** The state-of-the-art GaN-based α-particle detectors.

Affiliation	Type of Detector	DLT (μm)	Reverse Bias (V)	Source Energy (MeV)	Det. Energy (MeV)	CCE
Vilnius Univ. [36]	DSC	2–2.5	28	5.48	0.55	92%
Institute of Rare Metals [37]	Mesa	3	–	5.157	1.129	100%
Univ. of Glasgow [38]	Mesa	2.5	16	5.48	0.477	94%
		12	150	5.48	1.2	53%
Ohio State Univ. [34]	Sandwich	bulk	20	5.48	0.325	6%
Ohio State Univ. [35]	Sandwich	bulk	550	5.48	5.48	100%
Chonbuk National Univ. [33]	Sandwich	30/bulk	120	5.1	4.59	90%

**Table 4:** Material properties of MOCVD grown GaN drift layers (DL)s such as 2 theta-omega scan, root mean square (RMS) surface roughness, TDD, elemental concentrations and charge carrier density (CCD) measured by SIMS (Si limit 1.0 × 10^14^ and Mg limit 2.0 × 10^14^) and Hall.

	XRD (2 theta-omega scan)		TDD (MPPL) (× 10^6^/cm^2^)	RMS Roughness (AFM) (nm)	Si conc. (/cm^3^) (N_D_)	Mg conc. (/cm^3^) (N_A_)	CCD = N_D_−N_A_ (/cm^3^)	
DLT (μm)	002 (arc.sec)	102 (arc.sec)					SIMS	Hall
15	108.4	260.6	3.3	0.206	1.5 × 10^16^	1.5 × 10^16^	7.6 × 10^14^	7.5 × 10^14^
30	130	236	4.2	0.210	6 × 10^15^	1 × 10^15^	5 × 10^15^	5.2 × 10^15^

**Table 5:** List of state-of-the-art SBDs fabricated on GaN-on-GaN wafers.

Affiliation	DL (µm)	CCD (/cm^3^)	Barrier Height (eV)	Ideality Factor
NTU [49]	15	7.6 × 10^14^	0.81	1.03
	30	3 × 10^15^	0.78	1.3
Army Res. Labs Maryland [50,51]	6.6	3 × 10^16^	0.75	1.05
	2	2 × 10^16^	0.79	1.1
AIST Japan [52]	–	5 × 10^16^	0.74	1.09
Toyoda Gosei Co. [53]	10	2.2 × 10 ^16^	1.01	1.01
University of Fukui [54]	12	10^16^	1.05	1.03
Univ. of Notre Dame [29]	0.3	3 × 10^16^	1.1	1.4
